# Combining Metabolic Profiling and Gene Expression Analysis to Reveal the Biosynthesis Site and Transport of Ginkgolides in *Ginkgo biloba* L.

**DOI:** 10.3389/fpls.2017.00872

**Published:** 2017-05-26

**Authors:** Xu Lu, Hua Yang, Xinguang Liu, Qian Shen, Ning Wang, Lian-wen Qi, Ping Li

**Affiliations:** ^1^State Key Laboratory of Natural Medicines, China Pharmaceutical UniversityNanjing, China; ^2^Plant Biotechnology Research Center, School of Agriculture and Biology, Shanghai Jiao Tong UniversityShanghai, China

**Keywords:** *Ginkgo biloba*, metabolic profiling, gene expression, ginkgolide biosynthesis, transport

## Abstract

The most unique components of *Ginkgo biloba* extracts are terpene trilactones (TTLs) including ginkgolides and bilobalide. Study of TTLs biosynthesis has been stagnant in recent years. Metabolic profiling of 40 compounds, including TTLs, flavonoids, and phenolic acids, were globally analyzed in leaf, fibrous root, main root, old stem and young stem extracts of *G. biloba*. Most of the flavonoids were mainly distributed in the leaf and old stem. Most of phenolic acids were generally distributed among various tissues. The total content of TTLs decreased in the order of the leaf, fibrous root, main root, old stem and young stem. The TTLs were further analyzed in different parts of the main root and old stem. The content of TTLs decreases in the order of the main root periderm, the main root cortex and phloem and the main root xylem. In old stems, the content of TTLs in the cortex and phloem was much higher than both the old stem periderm and xylem. The expression patterns of five key genes in the ginkgolide biosynthetic pathway were measured by real-time quantitative polymerase chain reaction (RT-Q-PCR). Combining metabolic profiling and RT-Q-PCR, the results showed that the fibrous root and main root periderm tissues were the important biosynthesis sites of ginkgolides. Based on the above results, a model of the ginkgolide biosynthesis site and transport pathway in *G. biloba* was proposed. In this putative model, ginkgolides are synthesized in the fibrous root and main root periderm, and these compounds are then transported through the old stem cortex and phloem to the leaves.

## Introduction

The ‘living fossil’ *Ginkgo biloba*, which is a typical gymnosperm species with great economic and ecological values, has existed for more than 200 million years on Earth (Sabater-Jara et al., 2013; Zhang et al., 2015). The Ginkgo kernels have been used as traditional Chinese medicine for approximately 1000 years (He et al., 2015). In recent years, leaf extracts of *G. biloba*, which were developed as a standardized phytomedicine and named EGb761, have been widely used in the treatment of cardiovascular and neurological diseases (McKenna et al., 2001). The EGb761 is extracted from dried green leaves of *G. biloba*, which contain 6% terpene trilactones (TTLs), 24% flavonoids, and several phenolic acids (Drieu and Jaggy, 2000).

**FIGURE 1:**
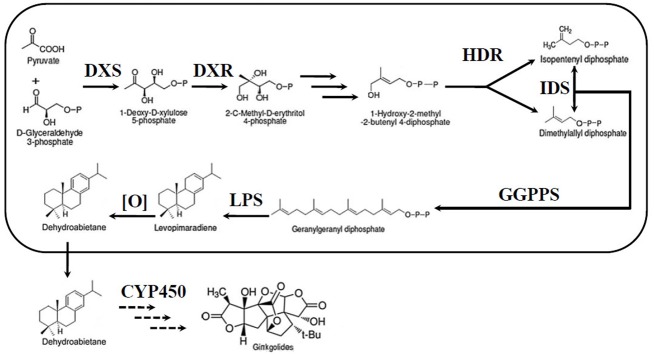
**Ginkgolide biosynthetic pathway**. DXS, DXP synthase; MEP, 2-C-methyl-D-erythritol-4-phosphate; DXR, DXP reductase; IDS, isopentenyl diphosphate isomerase; GGPPS, geranylgeranyl diphosphate synthase; HDR, 1-hydroxy-2-methyl-2-(E)-butenyl-4-diphosphate reductase; LPS, levopimaradiene synthase; CYP450, cytochrome P450-dependent oxidoreductase.

The most unique components of the extracts are the TTLs, including ginkgolides and bilobalide (Strømgaard and Nakanishi, 2004). Therefore, an increasing number of researchers have focused their studies on the TTLs of *G. biloba*. Despite their very complex structures, ginkgolides, and bilobalide are both biosynthesized from the basic isoprene units, dimethylallyl diphosphate (DMAPP) and isopentenyl diphosphate (IPP) (**Figure 1**). There are two independent pathways to produce IPP and DMAPP: the classic mevalonic acid (MVA) pathway and the methylerythritol phosphate (MEP) pathway (Banerjee and Sharkey, 2014; Pazouki and Niinemets, 2016). ^13^C-labeled glucose has been used to study the biosynthesis of ginkgolides. The results showed that ginkgolides are biosynthesized in the plastids through the MEP pathway (Schwarz and Arigoni, 1999). Recently, a series of genes in the MEP pathway, such as 1-deoxy-D-xylulose-5-phosphate synthase (DXS), 1-deoxy-D-xylulose-5-phosphate reductoisomerase (DXR) and isopentenyl diphosphate isomerase (IDS), were cloned in *G. biloba* (Gong et al., 2005; Gao et al., 2006; Kim et al., 2006a,b, 2008a,b; Lu et al., 2008). Under the catalysis of enzymes encoded by these genes, IPP and DMAPP were generated in the plastids. Three molecules of IPP and one molecule of DMAPP can synthesize the precursor of diterpenoids, geranylgeranyl diphosphate (GGPP), by geranylgeranyl diphosphate synthase (GGPPS) (Loto et al., 2012). Levopimaradiene synthase (LPS), a terpenoid synthase, catalyses GGPP cyclization to levopimaradiene, which is the first committed step in ginkgolide biosynthesis (Schepmann et al., 2001). The dehydrogenation of levopimaradiene produces dehydroabietane, which is transported into the cytoplasm (Sabater-Jara et al., 2013). Then, through a series of complex reactions including several cytochrome P450-dependent oxidoreductase (CYP450s) mediated oxidation steps and skeletal rearrangement, dehydroabietane is converted into ginkgolides (Schwarz and Arigoni, 1999). Compared with the ginkgolides biosynthetic pathway, the bilobalide biosynthetic pathway is not clear. As a sesquiterpene, some authors have suggested that bilobalide is most likely a product of partially degraded ginkgolide, but others have reported that bilobalide is derived from farnesyl diphosphate (FPP) (Peñuelas and Munné-Bosch, 2005; Dewick, 2009).

Although ginkgolides and bilobalide are mainly extracted from the leaves of *G. biloba*, the biosynthesis site of TTLs is not in the leaves. Both ^14^CO_2_ and (U-^14^C) glucose-labeled experiments in young seedlings of *G. biloba* showed that the biosynthesis of ginkgolides and bilobalide occurs in Ginkgo roots (Cartayrade et al., 1997; Neau et al., 1997). The *LPS* gene, the key gene of the ginkgolide biosynthetic pathway, has been cloned from *G. biloba*, and it has been found that the expression of *LPS* is predominantly detected in the root (Schepmann et al., 2001; Kim et al., 2012).

As early as 1997, the content of ginkgolide A (GA), ginkgolide B (GB), and bilobalide (BA) in *G. biloba* was separately measured in the root, stem, and leaf by HPLC (Cartayrade et al., 1997). The results showed that the content of GA, GB and BA was high in the roots and leavesbut low in the stems. Recently, GA, GB, ginkgolide C (GC), and BA were further measured in the cortex (bark) and xylem from roots and branches. The amount of TTLs in the cortex (bark) was 1.75–2.07-fold higher than that in the leaves (Liu et al., 2015). In this study, 40 compounds, including TTLs, flavonoids and phenolic acids were analyzed in leaf, fibrous root, main root, old stem and young stem of *G. biloba* by ultra-high-performance liquid chromatography coupled to triple-quadrupole mass spectrometry (UHPLC-QQQ-MS/MS). The content of terpene lactones was further analyzed in the main root periderm (R1), the main root cortex and phloem (R2), the main root xylem (R3), the old stem periderm (S1), the old stem cortex and phloem (S2) and the old stem xylem (S3). The expression patterns of five key genes in the ginkgolide biosynthetic pathway were analyzed by real-time quantitative polymerase chain reaction (RT-Q-PCR) in these tissues. Combining metabolic profiling and gene expression analysis further revealed the ginkgolide biosynthesis site and implied the transport of ginkgolides through the stem cortex and phloem (S2). This work provided important information of ginkgolide biosynthesis and transportation, and it will effectively promote the study of the ginkgolide biosynthetic pathway in the future.

## Materials and Methods

### Plant Materials

*Ginkgo biloba* plants from city of Pizhou, China, were grown in the Medicinal Botanical Garden of China Pharmaceutical University. All the tissues of *G. biloba* were collected in June 2015. After cleaning with distilled water, different tissues of *G. biloba* plants, including the main root, fibrous root, leaf, young stem and old stem, were collected. For the further analysis, the main root periderm (R1), the main root cortex and phloem (R2), the main root xylem (R3), the old stem periderm (S1), the old stem cortex and phloem (S2) and the old stem xylem (S3) were separated with a scalpel and tweezers under an anatomical lens. All the tissues were then immediately frozen in liquid nitrogen and stored in -80°C until used.

### Standards and Chemicals

Chromatographic methanol was obtained from Hanbon Tech Co. (Jiangsu Province, China). Acetonitrile of HPLC grade was purchased from TEDIA Company (Fairfield, OH, United States). Formic acid (99%, HPLC grade) was obtained from ROE (Newark, NC, United States). Deionized water was further purified with a Milli-Q water purification system (Millipore, Milford, MA, United States). Forty-two reference standards were purchased from the National Institute for the Control of Pharmaceutical and Biological Products (Beijing, China). Hesperidin and andrographolide were used as internal standards. All standards are listed in **Table 1**.

**Table 1:** The contents of each metabolite in different tissues.

Conc.(mg/g)	Metabolites	Leaves	Young stems	Old stems	Main roots	Fibrous roots
1	(-)-Epigallocatechin	0.002579 ± 0.000099	0.026386 ± 0.000833	0.012098 ± 0.001443	0.225474 ± 0.010343	0.110131 ± 0.010351
2	Protocatechuic acid	0.003144 ± 0.000454	0.807524 ± 0.113362	0.057343 ± 0.003283	0.157678 ± 0.007795	0.128197 ± 0.008732
3	*p*-Hydroxybenzoic acid	0.005995 ± 0.000425	0.000314 ± 0.000084	0.001342 ± 0.000237	0.000063 ± 0.000019	0.000773 ± 0.000072
4	Chlorogenic acid	0.000695 ± 0.000002	0.006317 ± 0.000236	0.001008 ± 0.000030	0.002305 ± 0.000228	0.000765 ± 0.000004
5	Catechin	0.016157 ± 0.000731	0.021558 ± 0.001290	0.090131 ± 0.001014	0.017431 ± 0.001077	0.004788 ± 0.000600
6	Caffeic acid (CA)	0.003023 ± 0.000559	0.189051 ± 0.032690	0.076542 ± 0.003885	0.020961 ± 0.000477	0.025137 ± 0.001045
7	Procyanidin B2	0.007597 ± 0.000254	0.000372 ± 0.000029	0.003142 ± 0.000567	0.000190 ± 0.000018	nd
8	Epicatechin	0.035722 ± 0.002166	0.002158 ± 0.000114	0.016350 ± 0.001827	0.001562 ± 0.000108	nd
9	p-Coumaric acid (p-coum)	0.000502 ± 0.000050	0.002307 ± 0.000130	0.000100 ± 0.000028	0.001164 ± 0.000112	0.001937 ± 0.000205
10	Bilobalide (BL)	3.117001 ± 0.210616	0.219511 ± 0.018890	1.231862 ± 0.132087	1.364673 ± 0.041411	1.293419 ± 0.110794
11	Ferulic acid	0.001020 ± 0.000115	0.007365 ± 0.000353	0.000986 ± 0.000302	0.004889 ± 0.000268	0.002294 ± 0.000340
12	Clitorin (KRRG)	0.350124 ± 0.032783	nd	0.000746 ± 0.000051	nd	nd
13	Ginkgolide J	0.097962 ± 0.011091	0.090293 ± 0.008029	0.089318 ± 0.007672	0.180703 ± 0.006699	0.156484 ± 0.009886
14	Quercetin-3-*O*-rutinoside (Rutin)	0.331425 ± 0.011372	nd	0.022529 ± 0.001271	0.000395 ± 0.000023	nd
15	Ginkgolide C	0.240238 ± 0.024936	0.380824 ± 0.027109	0.241639 ± 0.021113	0.568656 ± 0.023182	0.462576 ± 0.061852
16	(-)-Epicatechin gallate (ECG)	0.000026 ± 0.000004	0.000021 ± 0.000005	0.000022 ± 0.000011	0.000053 ± 0.000013	0.000019 ± 0.000007
17	Quercetin-3-*O*-β-D-glucoside (Q-3-G)	0.038616 ± 0.002516	0.002048 ± 0.000044	0.001701 ± 0.000187	0.000589 ± 0.000060	nd
18	Quercetin-3-*O*-β-D-glucopyranosyl-(1-2)-α-L-rhamnoside (QGR)	0.306884 ± 0.021763	0.000900 ± 0.000027	0.037349 ± 0.002731	0.000092 ± 0.000007	0.000149 ± 0.000023
19	Kaempferol-3-*O*-rutinoside (Kaem-3-RU)	0.250955 ± 0.006119	0.001216 ± 0.000069	0.012332 ± 0.001276	nd	nd
20	Isorhamnetin-3-*O*-rutinoside (Isor-3-RU)	0.215335 ± 0.010283	0.000893 ± 0.000026	0.074335 ± 0.006307	nd	nd
21	Quercetin-3-*O*-α-L-rhamnoside (Quer-3-R)	0.045818 ± 0.002422	nd	0.005285 ± 0.000276	nd	nd
22	Isorhamnetin-3-*O*-glucoside (Isor-3-G)	0.008899 ± 0.000701	nd	0.000682 ± 0.000055	nd	nd
23	Kaempferol-7-*O*-β-D-glucoside(Kaem-7-G)	0.007942 ± 0.000375	nd	nd	nd	nd
24	Apigenin-7-*O*-D-glucoside (Apig-7-G)	0.037659 ± 0.009918	nd	0.004509 ± 0.000970	nd	nd
25	Myricetin (Myri)	0.001872 ± 0.000125	0.001254 ± 0.000009	0.001236 ± 0.000017	0.001259 ± 0.000010	0.001281 ± 0.000012
26	Quercetin-3-*O*-α-L-rhamnopyranosyl-2”-(6”’-p-coumaroyl)-β-D-glucoside(QRCG)	1.383536 ± 0.201527	0.000330 ± 0.000037	0.385036 ± 0.028400	nd	nd
27	Kaempferol-3-*O*-α-L-rhamnopyranosyl-2”-(6”’-*p*-coumaroyl)-β-D-glucoside(KRCG)	1.483279 ± 0.166551	0.000364 ± 0.000065	0.039096 ± 0.004116	nd	nd
28	Ginkgolide A	0.840488 ± 0.086583	0.083398 ± 0.020813	0.475741 ± 0.051857	0.801193 ± 0.037190	1.282196 ± 0.203371
29	Ginkgolide B	0.504745 ± 0.048694	0.159157 ± 0.009136	0.420285 ± 0.024559	0.621048 ± 0.019988	0.535566 ± 0.048881
30	Luteolin	0.013845 ± 0.002020	nd	nd	nd	nd
31	Quercetin (Quer)	0.002397 ± 0.000329	0.000388 ± 0.000012	0.000168 ± 0.000012	0.000217 ± 0.000004	0.000091 ± 0.000075
32	Apigenin (Apig)	0.026858 ± 0.004593	nd	0.003751 ± 0.000382	nd	nd
33	Kaempferol (Kaem)	0.001139 ± 0.000200	0.000132 ± 0.000017	nd	nd	nd
34	Syringetin (Syri)	0.000095 ± 0.000019	0.000030 ± 0.000001	nd	0.000045 ± 0.000004	nd
35	Isorhamnetin (Isor)	0.001119 ± 0.000275	nd	nd	nd	nd
36	Amentoflavone	0.024378 ± 0.001374	0.001445 ± 0.000005	0.005380 ± 0.000444	nd	nd
37	Bilobetin	0.066267 ± 0.009192	0.002005 ± 0.000024	0.002698 ± 0.000133	0.000039 ± 0.000017	0.000012 ± 0.000001
38	Isoginkgetin	0.057700 ± 0.009321	0.004712 ± 0.000076	0.006041 ± 0.000334	nd	nd
39	Ginkgetin	0.226988 ± 0.030944	0.010164 ± 0.000084	0.008141 ± 0.000619	nd	nd
40	Sciadopitysin	0.066267 ± 0.009192	0.002005 ± 0.000024	0.002698 ± 0.000133	0.000039 ± 0.000017	0.000012 ± 0.000001

### Sample Extraction

The collected samples were homogenized in liquid nitrogen, and the powder from the samples was vacuum-lyophilized for 72 h. After weighing, the powder samples (100 mg/sample) were extracted with 70% aqueous methanol (8 mL) by vortexing for 3 min, followed by ultrasonication at room temperature for 60 min. Afterward, the resultant mixture was centrifuged at 13000 rpm for 10 min. The collected supernatant and 50-fold diluted supernatant were used for quantitation. All samples were filtered through 0.22 μm membrane filters before UHPLC-QQQ-MS/MS analysis.

### Metabolic Profiling Analysis in Different Tissues of *G. biloba* by UHPLC-QQQ-MS/MS

The samples were analyzed using a Shimadzu Nexera UHPLC with 8050 QQQ-MS/MS (Shimadzu, Japan). The method of metabolites analysis was performed as described previously with some modifications (Liu et al., 2014, 2015). The chromatographic separation was conducted with an Agilent Zorbax Extend C18 system equipped with a Narrow-bore column (2.1 mm× 150 mm, 5 μ, 80 A), with an injection volume of 2 μL. The sample vials were maintained at 4°C in a thermostatically controlled column compartment. The mobile phase consisted of 0.1% formic acid water (A) and acetonitrile (B). The mobile phases were programed with the gradient as follows: 0–5 min, 8–10% B; 5–15 min, 10–30% B; 15–20 min, 40–49% B; 20–23 min, 49% B; 23–25 min, 49–60% B; 25–26 min, 60–80% B; 26–28 min, 80% B; 28–30 min, 80–8% B; and 30–32 min, 8% B. The flow rate was 0.5 mL/min. Forty metabolites in *G. biloba* plants was quantitated in this work. A stock solution of 40 standard reference compounds was prepared by dissolving accurately weighted portions of the standards in methanol. Six different mixture solutions of standard concentration were then obtained by precisely mixing the 40 stock solutions with methanol. A calibration curve was constructed with at least six appropriate concentrations in triplicate. The regression equation and linear range of each metabolite are listed in Supplementary Table S1.

The ion source parameters of the mass spectrometer were as follows: nebulizing gas flow rate, 3 L/min; heating gas flow rate, 10 L/min; drying gas flow rate, 10 L/min; interface temperature, 300°C; desolvation temperature, 250°C; and heat block temperature, 500°C.

### Data Analysis

The raw data from the UHPLC-QQQ-MS/MS was quantitatively analyzed by the multiple reactions monitoring mode. The selection of precursor ion, product ion, collision energy (CE), and Q1/Q3 pre-bias were automatically optimized. All the parameters are listed in Supplementary Table S2. A heat map of the forty metabolites in *G. biloba* plants was constructed with the software MultiExperiment Viewer 4.9.0. For the statistical analysis, unsupervised principal component analysis (PCA) was used to further analyzed the metabolomic data using the software SIMCA-P 11.5 (Umetrics, Umea, Sweden). The amounts of forty reference compounds in each of the five samples (main root, fibrous root, leaf, young stem, and old stem) formed a data matrix with fifteen rows and forty columns, which was used for PCA analysis, after normalization. The first three principal components were extracted.

### Histological Analysis

Roots and stems of *G. biloba* were fixed in formalin-aceto-alcohol (FAA, 90 mL of 70% alcohol, 5 mL of 38% formaldehyde and 5 mL of glacial acetic acid) for 48 h. The fixed samples were sequentially dehydrated in 50, 70, 85, 95, and 100% ethanol. The tissues were then cleared, infiltrated and embedded in paraffin wax according to the methods of Ye et al. (2014). The sections of each tissue were made with an HM340E microtome (Microm International, Walldorf, Germany) and stained with safranine and Fast Green. The sections were observed with an Olympus BX60 microscope equipped with Sensys 1400E cooled charge-coupled device (CCD) camera.

### RT-Q-PCR Analysis

Total RNA was extracted from various tissues of *G. biloba* using a plant RNA isolation kit (BioTeke, Beijing, China) following the manufacturer’s instructions. All RNA samples were digested with DNase I (RNase-free) prior to use. Aliquots of 1 μg of total RNA were employed in the reverse transcriptase reaction using random hexamer primers for the synthesis of first-strand cDNA. The amplification reactions of RT-Q-PCR were performed on a LightCycler^®^ 96 Real-Time PCR System (Roche) with gene-specific primers, and the SYBR ExScript RT-PCR kit (Takara, Shiga, Japan) was used to confirm changes in gene expression. The thermal cycle conditions used were 5 min at 95°C followed by 40 cycles of amplification (15 s at 95°C, 15 s at 60°C and 30 s at 72°C). A melt curve analysis following each RT-Q-PCR was performed to assess product specificity. Expression patterns of ginkgolide biosynthesis key genes were analyzed in three biological replicates. Quantification of the target gene expression was carried out with comparative CT method (Livak and Schmittgen, 2001). The primers used in RT-Q-PCR are listed in Supplementary Table S3.

## Results

### Metabolic Profiling in Various Tissues of *G. biloba*

**FIGURE 2:**
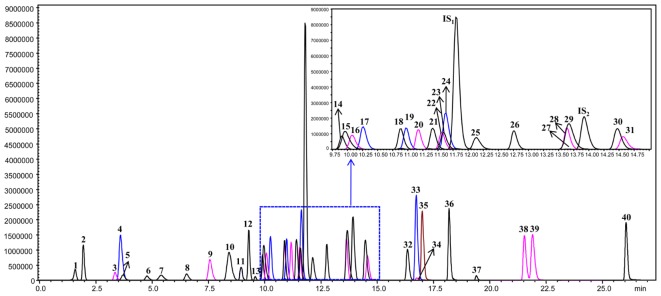
**The chromatograms of 40 compounds analyzed by UHPLC-QQQ-MS/MS in multiple reactions monitoring mode**. 1: (-)-Epigallocatechin; 2: Protocatechuic acid; 3: *p*-Hydroxybenzoic acid; 4: Chlorogenic acid; 5: Catechin; 6: Caffeic acid; 7: Procyanidin B2; 8: Epicatechin; 9: *p*-Coumaric acid; 10: Bilobalide; 11: Ferulic acid; 12: Clitorin; 13: Ginkgolide J; 14: Quercetin-3-*O*-rutinoside; 15: Ginkgolide C; 16: (-)-Epicatechin gallate; 17: Quercetin-3-*O*-β-D-glucoside; 18: Quercetin-3-O-β-D-glucopyranosyl-(1-2)-α-L-rhamnoside; 19: Kaempferol-3-*O*-rutinoside; 20: Isorhamnetin-3-*O*-rutinoside; 21: Quercetin-3-*O*-α-L-rhamnoside; 22: Isorhamnetin-3-*O*-glucoside; 23: Kaempferol-7-*O*-β-D-glucoside; 24: Apigenin-7-*O*-D-glucoside; 25: Myricetin; 26: Quercetin-3-*O*-α-L-rhamnopyranosyl-2”-(6”’-*p*-coumaroyl)-β-D-glucoside; 27: Kaempferol-3-*O*-α-L-rhamnopyranosyl-2”-(6”’-p-coumaroyl)-β-D-glucoside; 28: Ginkgolide A; 29: Ginkgolide B; 30: Luteolin; 31: Quercetin; 32: Apigenin; 33: Kaempferol; 34: Syringetin; 35: Isorhamnetin; 36: Amentoflavone; 37: Bilobetin; 38: Isoginkgetin; 39: Ginkgetin; 40: Sciadopitysin; IS1: Hesperidin; IS2: Andrographolide.

**FIGURE 3:**
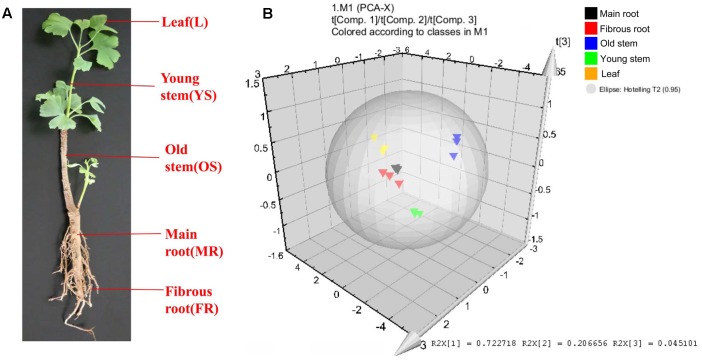
**Different tissues and PCA analysis in *Ginkgo biloba*. (A)** Different tissues of *G. biloba.* **(B)** PCA scores plot of 40 compounds in the leaf, young stem, old stem, main root, and fibrous root in *G. biloba*. Black triangles represent the main root, red triangles represent the fibrous root, blue triangles represent the old stem, green triangles represent the young stem and yellow triangles represent the leaf.

The leaf, young stem, old stem, main root, and fibrous root of *G. biloba* were analyzed by UHPLC-QQQ-MS/MS (**Figures 2**, **3A**). Forty compounds including terpene lactones, flavonoids and phenolic acids were identified by comparing the retention time and mass spectra with standards (Supplementary Table S2). **Figure 2** shows the chromatogram of forty mixed standards and internal standards (hesperidin and andrographolide). The content of these compounds was calculated according to the calibration curves with the internal standard method (Supplementary Table S1). PCA was used to further analyze the distribution and levels of metabolites in different tissues of *G. biloba*. As shown in **Figure 3B**, the results showed that the scores displayed in the PCA plot could be obviously divided into five relative clusters: leaf, young stem, old stem, main root, and fibrous root. There were significant differences in the occurrence and content of 40 compounds among different tissues of *G. biloba*.

**FIGURE 4:**
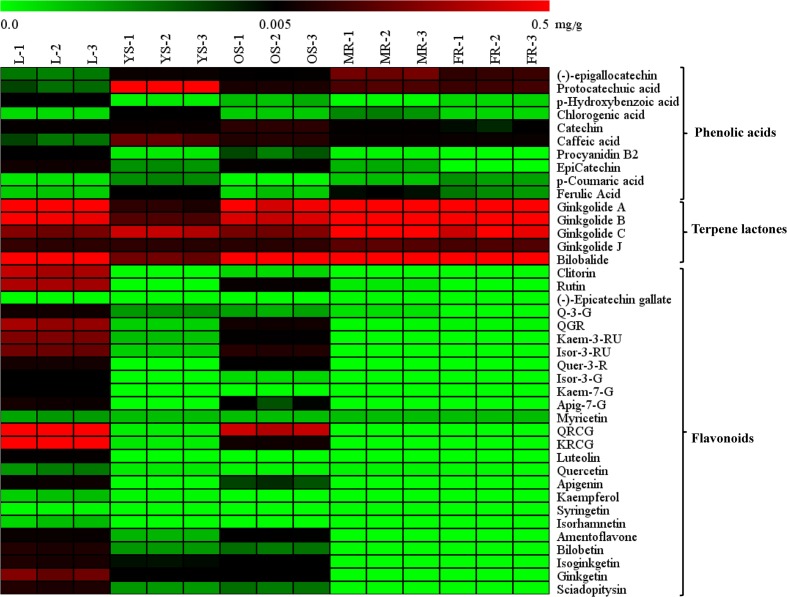
**Heat map of 40 compounds in the leaf, young stem, old stem, main root, and fibrous root of *G. biloba***. The contents of the compounds in different tissue samples were mapped and color-coded (green–black–red) by increasing the relative concentration.

The heat map (**Figure 4**) and **Table 1** shows the content of 40 detected compounds in different tissues of *G. biloba*. There are significant differences in the contents of flavonoids, terpene lactones and phenolic acids among different tissues of *G. biloba*. Most of the flavonoids were mainly distributed in aerial parts especially in the leaves and old stems. Only myricetin, (-)-epicatechin gallate (ECG) and quercetin were generally distributed in the various tissues. Most of phenolic acids were generally distributed among the various tissues of *G. biloba*. Only protocatechuic acid, chlorogenic acid, and caffeic acid (CA) were mainly distributed in the young stems of *G. biloba*. Terpene lactones, which include ginkgolides and bilobalide, were widely distributed in various tissues of *G. biloba*, and the content in the leaves and roots was much higher. The total content of terpene lactones decreases in the order of the leaf, fibrous root, main root, old stem, and young stem.

### Combined Metabolic Profiling Analysis and Expression Patterns of Key Ginkgolide Biosysthesis Genes in Various Tissues of *G. biloba*

**FIGURE 5:**
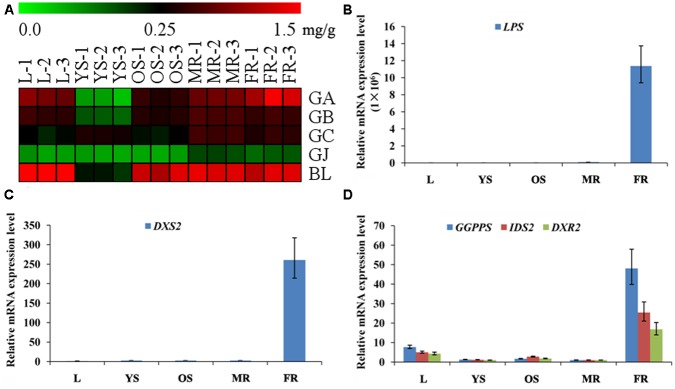
**Heat map of terpene trilactones (TTLs) in different tissues of *G. biloba* and expression analysis of the key ginkgolide biosynthetic pathway genes by real-time quantitative polymerase chain reaction (RT-Q-PCR). (A)** Heat map of terpene trilactones (TTLs) in the leaf, young stem, old stem, main root, and fibrous root of *G. biloba*. **(B)** The expression pattern of *LPS* in different tissues of *G. biloba.* **(C)** The expression pattern of *DXS2* in different tissues of *G. biloba.* **(D)** The expression patterns of *GGPPS*, *DXR2*, *and IDS2* in different tissues of *G. biloba.*

Among the metabonomics data, the terpene lactones including GA, GB, GC, ginkgolide J (GJ), and BA were further analyzed (**Figure 5A**). The content of GA, GB, GC, and BA were much higher than that of GJ in various tissues of *G. biloba*. The content of GA was the highest in the fibrous root, while other ginkgolides were highest in the main root. The distribution of bilobalide was unique, being highest in the leaf.

To reveal the relationship of terpene lactone distribution and biosynthesis, the expression patterns of five key genes in the ginkgolide biosynthetic pathway were analyzed by RT-Q-PCR. The results showed that *GbLPS* is mainly expressed in the fibrous root (**Figure 5B**). In addition, *GbDXS2* was highly expressed in fibrous roots, which showed nearly 260-fold higher expression compared to other tissues (**Figure 5C**). The expression patterns of *GbDXR2*, *GbIDS2*, and *GbGGPPS* were similar to that of *GbDXS2* (**Figure 5D**). The combined the metabonomics and expression analysis results show that the fibrous root is the main biosynthesis site of ginkgolides.

### Further Analysis of Terpene Lactone Distribution and Expression Patterns of Key Ginkgolide Biosynthesis Genes in Various Parts of Roots

**FIGURE 6:**
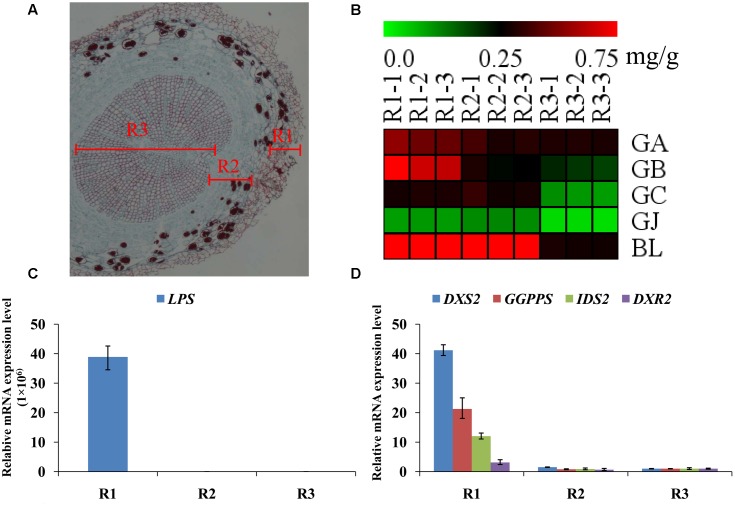
**Microstructure of the main root, heat map of TTLs in different parts of the *G. biloba* main root and expression analysis of the key ginkgolide biosynthetic pathway genes by RT-Q-PCR. (A)** Paraffin section of the main root. Three parts were clearly identified. **(B)** Heat map of TTL content in the main root periderm (R1), the main root cortex and phloem (R2) and the main root xylem (R3) of *G*. *biloba*. **(C)** The expression pattern of *LPS* in different parts of the main root. **(D)** The expression patterns of *DXS2*, *GGPPS*, *DXR2*, *and IDS2* in different parts of the main root.

The main root was divided into three parts the main root periderm (R1), the main root cortex and phloem (R2) and the main root xylem (R3) for further metabolic analysis and expression analyses. The content of TTLs among R1, R2 and R3 showed significant differences (**Figure 6A** and **Table 2**). GA, GB, and BL, the combined content of which accounted for >85% of TTLs, exhibited the highest content in R1. In addition, the content of GB in R1 is 2.5-fold and 3.4-fold higher than that of R2 and R3, respectively, while the distribution of GC and GJ were different from that of the above mentioned TTLs. GC and GJ displayed the highest content in R2 (**Figure 6B**).

**Table 2:** The content of TTLs in different parts of old stems and main roots.

Metabolites (mg/g)	R1	R2	R3	S1	S2	S3
GA	0.485146 ± 0.043369	0.340316 ± 0.042308	0.311272 ± 0.006338	0.126904 ± 0.000817	0.593898 ± 0.010968	0.131427 ± 0.008101
GB	0.677990 ± 0.062542	0.267641 ± 0.036986	0.199695 ± 0.013680	0.133744 ± 0.021552	0.945785 ± 0.013968	0.098547 ± 0.005608
GC	0.302886 ± 0.009992	0.313549 ± 0.037445	0.102043 ± 0.007530	0.094636 ± 0.016559	0.818323 ± 0.012528	0.076334 ± 0.003117
GJ	0.097531 ± 0.002758	0.114819 ± 0.002620	0.034631 ± 0.002814	0.033916 ± 0.002348	0.273677 ± 0.002346	0.018170 ± 0.001054
BL	1.082255 ± 0.046603	0.782537 ± 0.014354	0.291869 ± 0.009781	0.410405 ± 0.000392	0.628000 ± 0.011084	0.179076 ± 0.004623

Real-time quantitative polymerase chain reaction was used to detect expression of the key gene of the ginkgolide biosynthetic pathway. The results showed that *GbLPS* is specifically expressed in R1, which implies that the site of ginkgolides biosynthesis should be in R1 (**Figure 6C**). The expression of *GbDXS2*, *GbDXR2* and both *GbIDS2* and *GbGGPPS* was also detected, respectively, in R1, R2, and R3. In addition, the expression levels of these four genes were significantly higher in R1 (**Figure 6D**).

### Further Analysis of TTL Distribution in Various Parts of Stems

**FIGURE 7:**
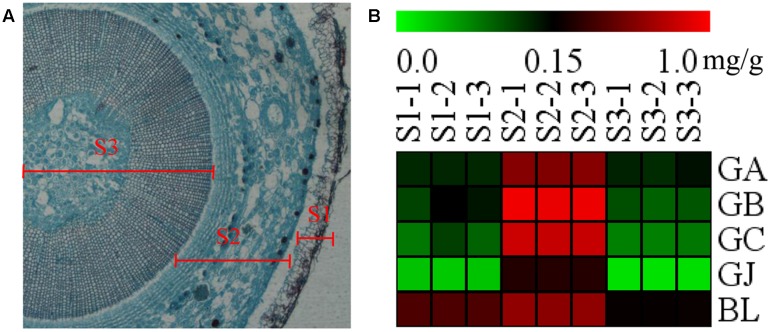
**Microstructure of the stem and heat map of TTLs in different parts of *G. biloba* stem. (A)** Paraffin section of the old stem. **(B)** Heat map of TTL content in the old stem periderm (S1), the old stem cortex and phloem (S2), and the old stem xylem (S3) of *G*. *biloba*.

The stem was divided into three parts, the old stem periderm (S1), the old stem cortex and phloem (S2) and the old stem xylem (S3) for further metabolic analysis by UHPLC-QQQ-MS/MS (**Figure 7A**). The distribution of TTLs in the stem was significantly different from that in the root. The GA, GB, GC, GJ, and BL in the stem had the highest content in S2. The total content of TTLs decreased in the order of S2, S1, and S3, with S2 showing a 4-fold and 6.5-fold higher total TTL content than S1 and S3, respectively (**Figure 7B** and **Table 2**). The most abundant TTL in S2 was GB, followed by GC, BL, GA, and GJ.

## Discussion

### Fibrous Root Is the Important Biosynthesis Site of Ginkgolides

Previous studies showed that the synthesis of ginkgolides was mainly in the root of *G. biloba*, but it is not clear in which part of the root ginkgolides are synthesized. To further analyze the synthesis site of ginkgolides, *G. biloba* plants were divided into five parts (including the main root, fibrous root, leaf, young stem, and old stem) for quantitative analysis. The metabolic profiling results showed that the content of TTLs followed the order of the leaf > fibrous root > main root > old stem > young stem. The expression analysis showed that the key genes in the ginkgolide biosynthetic pathway were highly expressed in the fibrous root, while the expression levels of these genes were very low or even undetectable in the mainroot and other aerial portions. Our results demonstrated that the fibrous root is the important biosynthesis site of ginkgolides.

### Main Root Periderm Is Another Ginkgolide Biosynthesis Site in *G. biloba*

We further analyzed the metabolites and gene expression in different parts of main root. The main root of *G. biloba* was divided into three parts, including the main root periderm (R1), the main root cortex and phloem (R2) and the main root xylem (R3). The results of the metabolic analysis showed that GA and GB have the highest content in R1. The content of GA and GB was in the order of R1 > R2 > R3. GC and GJ displayed the highest content in R2. The expression analysis results showed that the ginkgolide biosynthetic pathway key gene *GbLPS* is specifically expressed in the main root periderm (R1). *GbDXS2*, *GbDXR2*, *GbIDS2* and *GbGGPPS* were highly expressed in R1, with little expression in R2 and R3. Previous studies showed that GA is the first compound in the ginkgolide biosynthetic pathway. GB, GC, and GJ are the derivatives of GA by successive additions of a hydroxyl group (Cartayrade et al., 1997; Schafer, 2015). Combining the above results has demonstrated that the main root periderm (R1) is also an important ginkgolides synthesis site of *G. biloba*. The results were similar with those in *Salvia miltiorrhi*za Bunge, a widely used herb in traditional Chinese medicine. The key genes of the tanshinone biosynthetic pathway are mainly expressed in the root periderm of *Salvia miltiorrhiza* and tanshinone production is also mainly carried out in the root periderm (Xu et al., 2015).

### Terpene Lactones Move to the Leaves Through the Stem Cortex and Phloem

The key genes of the ginkgolide biosynthetic pathway have only slight or even no expression in the stem of *G. biloba*. When combining our results with those the previous studies, we know that there is no ginkgolide biosynthesis in the stem of *G. biloba* (Cartayrade et al., 1997; Neau et al., 1997; Kim et al., 2012). Cartayrade et al. (1997) reported that after ^14^CO_2_ labeling experiments, the labeled ginkgolides were first detected in roots and subsequently in the stems and leaves. *GbLPS* promoter-driven GUS expression in transgenic Arabidopsis alluded that the translocation of ginkgolides occurs through the phloem (Kim et al., 2012).

**FIGURE 8:**
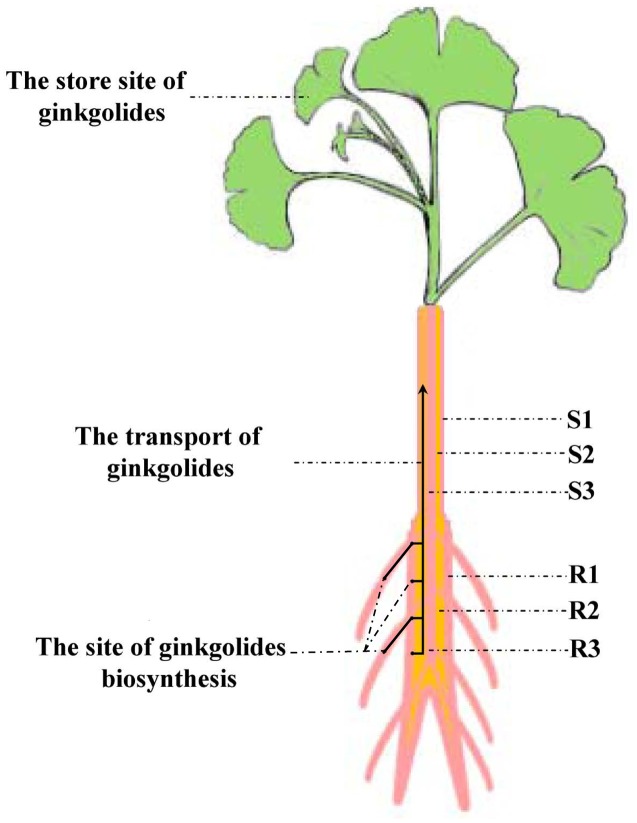
**Putative model of ginkgolide biosynthesis site and transport pathway**. R1, main root periderm; R2, main root cortex and phloem; R3, main root xylem; S1, old stem periderm; S2, old stem cortex and phloem; S3, old stem xylem.

Our experimental results showed that the content of ginkgolides and bilobalide in the cortex and phloem of old stem (S2) is much higher than that in the old stem periderm (S1) and old stem xylem (S3) in *G. biloba*. These results imply that ginkgolides and bilobalide can be transported from the roots to the leaves via S2. Several secondary compounds, such as iridoid glycosides, pyrrolizidine alkaloids and glucosinolates, can also be transported in the phloem (Robert and Shmuel, 2009). In *Senecio vernalis*, synthesis of the backbone structure of the pyrrolizidine alkaloid (PA) is restricted to the roots. PAs can move from the roots to shoots via the phloem (Moll et al., 2002). Here, our results first indicate that ginkgolides in *G. biloba* are produced in the fibrous root and root periderm of the main root, and then move to leaves though the cortex and phloem of the old stem (S2) (**Figure 8**). In addition, leaves seem to act as a storage location of ginkgolides and bilobalide only (**Figure 8**).

## Conclusion

The metabolics profile of forty compounds, including terpene lactones, flavonoids and phenolic acids, was analyzed in different tissues of *G. biloba* by UHPLC-QQQ-MS/MS. The content of terpene lactones was further analyzed in the main root periderm (R1), the main root cortex and phloem (R2), the main root xylem (R3), the old stem periderm (S1), the old stem cortex and phloem (S2) and the old stem xylem (S3). The expression patterns of five key genes in the ginkgolide biosynthetic pathway showed that the key genes are mainly expressed in the fibrous root and main root periderm (R1). The combined metabolic profiling and RT-Q-PCR results revealed that ginkgolides are synthesized in the fibrous root and main root periderm (R1) of *G. biloba* and that ginkgolides could move to the leaves through the cortex and phloem of the old stem tissue (S2). Our study provides useful information for the study of ginkgolide biosynthesis, and our results will effectively promote the discovery of new key genes involved in the ginkgolides biosynthetic pathway in future.

## Author Contributions

XL, L-wQ, and PL designed the research; XGL, HY, and XL performed experiments with metabolic profiling analyses and gene expression analyses. XL, QS, and NW wrote and revised the article.

## Conflict of Interest Statement

The authors declare that the research was conducted in the absence of any commercial or financial relationships that could be construed as a potential conflict of interest.
