# Supplementary material for: Combining Metabolic Profiling and Gene Expression Analysis to Reveal the Biosynthesis Site and Transport of Ginkgolides in Ginkgo biloba L
Source: Front Plant Sci. 2017 May 26;8:872. doi: 10.3389/fpls.2017.00872 (PMC5445427; doi:10.3389/fpls.2017.00872)
Supplement: Supplementary file 1 [file Table_1.DOCX]

**Supplementary table 1** The regression equation and linear range of each metabolite

|  | Metabolites | Regression equation | R^2^ | Linear range (µg/mL) | |
| --- | --- | --- | --- | --- | --- |
| 1 | (-)-Epigallocatechin | y = 0.0369 x - 0.0002 | 0.9992 | 0.00025-2.5 |  |
| 2 | Protocatechuic acid | y = 0.0880 x - 0.0001 | 0.9992 | 0.00025-2.5 |  |
| 3 | p-Hydroxybenzoic acid | y = 0.0417 x + 0.0007 | 0.9991 | 0.00025-2.5 |  |
| 4 | Chlorogenic acid | y = 0.1665 x - 0.0061 | 0.9998 | 0.00025-10 |  |
| 5 | Catechin | y = 0.0213 x + 0.00002 | 0.9998 | 0.00025-2.5 |  |
| 6 | Caffeic acid (CA) | y = 0.0298 x - 0.0003 | 0.9991 | 0.00025-2.5 |  |
| 7 | Procyanidin B2 | y = 0.0297 x - 0.0002 | 0.9999 | 0.00025-2.5 |  |
| 8 | Epicatechin | y = 0.0399 x - 0.0001 | 1.0000 | 0.00025-2.5 |  |
| 9 | p-Coumaric acid (p-coum) | y = 0.1009 x + 0.0006 | 0.9999 | 0.00025-2.5 |  |
| 10 | Bilobalide (BL) | y = 0.2944 x + 0.0011 | 0.9996 | 0.00025-2.5 |  |
| 11 | Ferulic Acid | y = 0.0107 x - 0.0001 | 0.9996 | 0.00025-5 |  |
| 12 | Clitorin(KRRG) | y = 0.1051 x - 0.0008 | 0.9998 | 0.00025-2.5 |  |
| 13 | Ginkgolide J | y = 0.0079 x - 0.0001 | 0.9994 | 0.00025-2.5 |  |
| 14 | Quercetin-3-O-rutinoside (Rutin) | y = 0.0433 x - 0.0002 | 1.0000 | 0.00025-2.5 |  |
| 15 | Ginkgolide C | y = 0.3392 x -0.0025 | 1.0000 | 0.00025-2.5 |  |
| 16 | (-)-Epicatechin gallate (ECG) | y = 0.0580 x - 0.0001 | 0.9997 | 0.00025-10 |  |
| 17 | Quercetin-3-O-β-D-glucoside (Q-3-G) | y = 0.1100 x - 0.0016 | 0.9990 | 0.00025-2.5 |  |
| 18 | Quercetin-3-O-β-D-glucopyranosyl-(1-2)-α-L-rhamnoside (QGR) | y = 0.0949 x - 0.0009 | 0.9994 | 0.00025-5 |  |
| 19 | Kaempferol-3-O-rutinoside (Kaem-3-RU) | y = 0.1267 x - 0.0015 | 0.9994 | 0.00025-2.5 |  |
| 20 | Isorhamnetin-3-O-rutinoside (Isor-3-RU) | y = 0.0874 x - 0.0006 | 0.9999 | 0.00025-2.5 |  |
| 21 | Quercetin-3-O-α-L-rhamnoside (Quer-3-R) | y = 0.0826 x - 0.00004 | 0.9992 | 0.00025-5 |  |
| 22 | Isorhamnetin-3-O-glucoside (Isor-3-G) | y = 0.0854 x + 0.0002 | 0.9992 | 0.00025-5 |  |
| 23 | Kaempferol-7-O-β-D-glucoside(Kaem-7-G) | y = 0.0919 x - 0.0002 | 0.9994 | 0.00025-2.5 |  |
| 24 | Apigenin-7-O-D-glucoside (Apig-7-G) | y = 0.1460 x - 0.0009 | 0.9998 | 0.00025-2.5 |  |
| 25 | Myricetin (Myri) | y = 0.0735 x - 0.0005 | 0.9997 | 0.00025-2.5 |  |
| 26 | Quercetin-3-O-α-L-rhamnopyranosyl-2''-(6'''-p-coumaroyl)-β-D-glucoside(QRCG) | y = 0.0911 x + 0.0008 | 0.9995 | 0.00025-2.5 |  |
| 27 | Kaempferol-3-O-α-L-rhamnopyranosyl-2''-(6'''-p-coumaroyl)-β-D-glucoside(KRCG) | y = 0.0632 x + 0.0003 | 0.9998 | 0.00025-10 |  |
| 28 | Ginkgolide A | y = 0.0062 x -0.00002 | 0.9991 | 0.00025-2.5 |  |
| 29 | Ginkgolide B | y = 0.6640 x - 0.0076 | 0.9997 | 0.00025-10 |  |
| 30 | Luteolin | y = 0.1577 x - 0.0002 | 1.0000 | 0.00025-2.5 |  |
| 31 | Quercetin (Quer) | y = 0.0911 x + 0.0008 | 0.9995 | 0.00025-2.5 |  |
| 32 | Apigenin (Apig) | y = 0.1203 x + 0.0032 | 0.9993 | 0.00025-2.5 |  |
| 33 | Kaempferol (Kaem) | y = 0.0106 x + 0.0001 | 0.9994 | 0.00025-2.5 |  |
| 34 | Syringetin (Syri) | y = 0.3167 x + 0.0012 | 0.9996 | 0.00025-2.5 |  |
| 35 | Isorhamnetin (Isor) | y = 0.2734 x + 0.0020 | 0.9996 | 0.00025-2.5 |  |
| 36 | Amentoflavone | y = 0.2061 x + 0.0013 | 0.9987 | 0.00025-1 |  |
| 37 | Bilobetin | y = 0.1577 x - 0.0002 | 1.0000 | 0.00025-2.5 |  |
| 38 | Isoginkgetin | y = 0.0911 x + 0.0008 | 0.9995 | 0.00025-2.5 |  |
| 39 | Ginkgetin | y = 0.1203 x + 0.0032 | 0.9993 | 0.00025-2.5 |  |
| 40 | Sciadopitysin | y = 0.0106 x + 0.0001 | 0.9994 | 0.00025-2.5 |  |
