# Supplementary material for: Combining Metabolic Profiling and Gene Expression Analysis to Reveal the Biosynthesis Site and Transport of Ginkgolides in Ginkgo biloba L
Source: Front Plant Sci. 2017 May 26;8:872. doi: 10.3389/fpls.2017.00872 (PMC5445427; doi:10.3389/fpls.2017.00872)
Supplement: Supplementary file 2 [file Table_2.DOCX]

**Supplementary table 2** The MS parameters of each metabolite

|  | Metabolites | Retention time (s) | Precursor *m/z* | Product *m/z* | | | Q1 Pre Bias (V) | CE | Q3 Pre Bias (V) |
| --- | --- | --- | --- | --- | --- | --- | --- | --- | --- |
| 1 | (-)-Epigallocatechin | 1.52 | 305.1 | | 125 | 14 | | 23 | 23 |
| 2 | Protocatechuic acid | 1.88 | 153 | | 109.05 | 30 | | 15 | 20 |
| 3 | p-Hydroxybenzoic acid | 3.22 | 137.1 | | 92.95 | 28 | | 17 | 17 |
| 4 | Chlorogenic acid | 3.4 | 353.2 | | 191.05 | 25 | | 14 | 19 |
| 5 | Catechin | 3.49 | 289.2 | | 245 | 20 | | 14 | 26 |
| 6 | Caffeic acid (CA) | 4.53 | 179.1 | | 134.05 | 12 | | 25 | 25 |
| 7 | Procyanidin B2 | 5.03 | 577.1 | | 407.05 | 20 | | 24 | 28 |
| 8 | Epicatechin | 6.2 | 289.1 | | 245.05 | 20 | | 15 | 26 |
| 9 | p-Coumaric acid (p-coum) | 7.53 | 163.1 | | 119.05 | 11 | | 16 | 22 |
| 10 | Bilobalide (BL) | 8.38 | 325.2 | | 191.05 | 23 | | 17 | 30 |
| 11 | Ferulic Acid | 8.82 | 193.1 | | 134.05 | 13 | | 18 | 25 |
| 12 | Clitorin(KRRG) | 9.31 | 739.2 | | 284.05 | 20 | | 45 | 29 |
| 13 | Ginkgolide J | 9.48 | 423.1 | | 349.05 | 20 | | 27 | 23 |
| 14 | Quercetin-3-O-rutinoside (Rutin) | 9.75 | 609.10 | | 300.10 | 20 | | 38 | 30 |
| 15 | Ginkgolide C | 9.79 | 439.20 | | 383.10 | 21 | | 15 | 26 |
| 16 | (-)-Epicatechin gallate (ECG) | 9.89 | 441.10 | | 169.00 | 10 | | 20 | 17 |
| 17 | Quercetin-3-O-β-D-glucoside (Q-3-G) | 10.12 | 463.10 | | 300.05 | 10 | | 27 | 20 |
| 18 | Quercetin-3-O-β-D-glucopyranosyl-(1-2)-α-L-rhamnoside  (QGR) | 10.73 | 609.20 | | 300.05 | 20 | | 37 | 30 |
| 19 | Kaempferol-3-O-rutinoside (Kaem-3-RU) | 10.83 | 593.20 | | 285.05 | 20 | | 32 | 30 |
| 20 | Isorhamnetin-3-O-rutinoside (Isor-3-RU) | 11.04 | 623.20 | | 315.10 | 20 | | 31 | 21 |
| 21 | Quercetin-3-O-α-L-rhamnoside (Quer-3-R) | 11.24 | 447.10 | | 300.00 | 10 | | 26 | 20 |
| 22 | Isorhamnetin-3-O-glucoside (Isor-3-G) | 11.41 | 477.10 | | 314.00 | 10 | | 28 | 20 |
| 23 | Kaempferol-7-O-β-D-glucoside(Kaem-7-G) | 11.44 | 447.10 | | 285.00 | 12 | | 24 | 30 |
| 24 | Apigenin-7-O-D-glucoside (Apig-7-G) | 11.47 | 431.10 | | 268.00 | 10 | | 32 | 28 |
| 25 | Myricetin (Myri) | 11.98 | 317.10 | | 151.00 | 11 | | 24 | 30 |
| 26 | Quercetin-3-O-α-L-rhamnopyranosyl-2''-(6'''-p-coumaroyl)-β-D-glucoside(QRCG) | 12.62 | 755.00 | | 300.10 | 20 | | 47 | 30 |
| 27 | Kaempferol-3-O-α-L-rhamnopyranosyl-2''-(6'''-p-coumaroyl)-β-D-glucoside(KRCG) | 13.47 | 739.20 | | 284.05 | 20 | | 47 | 30 |
| 28 | Ginkgolide A | 13.53 | 407.20 | | 351.10 | 30 | | 16 | 25 |
| 29 | Ginkgolide B | 13.53 | 423.10 | | 367.05 | 29 | | 15 | 23 |
| 30 | Luteolin | 14.31 | 285.10 | | 133.05 | 10 | | 33 | 24 |
| 31 | Quercetin (Quer) | 14.38 | 301.10 | | 150.95 | 10 | | 21 | 28 |
| 32 | Apigenin (Apig) | 16.2 | 269.00 | | 117.05 | 12 | | 34 | 22 |
| 33 | Kaempferol (Kaem) | 16.64 | 285.00 | | 93.00 | 10 | | 35 | 17 |
| 34 | Syringetin (Syri) | 16.66 | 345.10 | | 315.00 | 12 | | 26 | 21 |
| 35 | Isorhamnetin (Isor) | 16.87 | 315.10 | | 299.95 | 10 | | 20 | 13 |
| 36 | Amentoflavone | 18.18 | 537.10 | | 375.05 | 20 | | 32 | 25 |
| 37 | Bilobetin | 19.34 | 553.10 | | 521.00 | 40 | | 31 | 36 |
| 38 | Isoginkgetin | 21.51 | 565.10 | | 533.05 | 20 | | 28 | 38 |
| 39 | Ginkgetin | 21.87 | 567.20 | | 535.10 | 20 | | 29 | 38 |
| 40 | Sciadopitysin | 25.51 | 579.20 | | 547.05 | 28 | | 27 | 26 |
| IS1 | Hesperidin | 11.68 | 609.1 | | 301.1 | 22 | | 25 | 20 |
| IS2 | Andrographolide | 13.78 | 395.2 | | 331.15 | 28 | | 12 | 22 |
