# Supplementary material for: Combining Metabolic Profiling and Gene Expression Analysis to Reveal the Biosynthesis Site and Transport of Ginkgolides in Ginkgo biloba L
Source: Front Plant Sci. 2017 May 26;8:872. doi: 10.3389/fpls.2017.00872 (PMC5445427; doi:10.3389/fpls.2017.00872)
Supplement: Supplementary file 3 [file Table_3.DOCX]

**Supplementary table 3** Primers used for RT-PCR in this study

| Primers Primer Sequence(5’-3’) |
| --- |
| GbLPS-RT-F CAGCACTGCGTCCGTTCTCACTT  GbLPS-RT-R CATGCACTGGATTTCCTTCACCAATGTCT  GbGGPPS-RT-F TTTGAGAAAGGGGTTACTGTCGTCCAG  GbGGPPS-RT-R TTCTCAGGGTAATGCGTAGGAACAGC  GbIDS2-RT-F GATGCTACTCAGGAAAGACAAGATGCAATG  GbIDS2-RT-R GTCCAATACGCTCCTTAGAGTCAATCC  GbDXS2-RT-F GCATTTTACAAGAAGGAAGCCGAGTAGCAA  GbDXS2-RT-R GCCAACTGCCTAATCAAATCTCCATCC  GbDXR2-RT-F GCACACTACCTTTATGGGGCTGACTA  GbDXR2-RT-R CCGACCAGCAGAATAAGCAAGATCCA  Gb18S-RT-F ATAACTCGACGGATCGCACGGC  Gb18S-RT-R CCTTCCTTGGATGTGGTAGCCG |
